# Psychological factors that promote behavior modification by obese patients

**DOI:** 10.1186/1751-0759-3-9

**Published:** 2009-09-25

**Authors:** Hitomi Saito, Yutaka Kimura, Sawako Tashima, Nana Takao, Akinori Nakagawa, Takanobu Baba, Suguru Sato

**Affiliations:** 1 Center for Studies on Emotions, Stress, and Health, Doshisha University, Karasuma-Higashi Imadegawa, Kamigyo-ku Kyoto-shi, Kyoto 602-8580, Japan; 2 Health Science Center, Kansai Medical University, 3-1 Shinmachi 2-chome, Hirakata-shi, Osaka 573-1191, Japan; 3 Graduate School of Letters, Doshisha University, Karasuma-Higashi Imadegawa, Kamigyo-ku Kyoto-shi, Kyoto 602-8580, Japan; 4 Otemon Gakuin University, 1-15 Nishiai 2-chome, Ibaraki, Osaka 567-8502, Japan; 5 Faculty of Psychology, Doshisha University, Karasuma-Higashi Imadegawa, Kamigyo-ku Kyoto-shi, Kyoto 602-8580, Japan

## Abstract

**Background:**

The weight-loss effect of team medical care in which counseling is provided by clinical psychologists was investigated in an university hospital obesity (OB) clinic. Nutritional and exercise therapy were also studied. In our previous study, we conducted a randomized, controlled trial with obese patients and confirmed that subjects who received counseling lost significantly more weight than those in a non-counseling group. The purpose of this study was to identify the psychological characteristics assessed by ego states that promote behavior modification by obese patients.

**Methods:**

147 obese patients (116 females, 31 males; mean age: 45.9 ± 15.4 years) participated in a 6-month weight-loss program in our OB clinic. Their psychosocial characteristics were assessed using the Tokyo University Egogram (TEG) before and after intervention. The Wilcoxon signed rank test was used to compare weight and psychological factors before and after intervention. Multiple regression analysis was used to identify factors affecting weight loss.

**Results:**

Overall, 101 subjects (68.7%) completed the program, and their data was analyzed. The subjects mean weight loss was 6.2 ± 7.3 kg (*Z *= 7.72, *p *< 0.01), and their mean BMI decreased by 2.4 ± 2.7 kg/m^2 ^(*Z *= 7.65, *p *< 0.01). Significant differences were observed for the Adult (A) ego state (0.68 ± 3.56, *Z *= 1.95, *p *< 0.05) and the Free Child (FC) ego state (0.59 ± 2.74, *Z *= 2.46, *p *< 0.01). The pre-FC ego state had a significant effect on weight loss (β = 0.33, *p *< 0.01), and a tendency for changes in the A ego state scores to affect weight loss (β = - 0.20, *p *= 0.06) was observed.

**Conclusion:**

This study of a 6-month weight-loss program that included counseling by clinical psychologists confirmed that the A ego state of obese patients, which is related to their self-monitoring skill, and the FC ego state of them, which is related to their autonomy, were increased. Furthermore, the negative aspects of the FC ego state related to optimistic and instinctive characteristics inhibited the behavior modification, while the A ego state represented objective self-monitoring skills that may have contributed to weight loss.

## Background

Behavior modification is essential for the prevention and treatment of obesity, which is one of the critical risk factors for lifestyle-related diseases. However, it is extremely difficult to encourage people to modify their behavior in order to achieve a healthier lifestyle because lifestyles largely depend on individual beliefs and values. For this reason, a behavioral scientific approach would be helpful [1]. It is also important to note that lifestyle-related diseases are closely associated with psychosocial stress [2]. Thus, it is necessary to assess obese patients' psychosocial status and to provide them with psychological support.

Given this background, we started an obesity clinic (OB clinic) in 1999. In the OB clinic, medical doctors, clinical psychologists, registered dietitians, and exercise trainers support obese people, providing team medical care [3,4]. Clinical psychologists assess the patients' psychosocial situation and identify factors that could prevent weight loss. We also try to encourage patients to change their lifestyles by themselves, by maintaining a dialogic relationship aimed at enhancing their self-effectiveness and autonomy. As part of this "team medical care", staff members share patient information and provide comprehensive treatment.

In our previous study, we conducted a randomized, controlled trial with obese patients between 1999 and 2001. In that study, weight was significantly reduced by patients who received counseling from clinical psychologists, as well as nutritional and exercise therapy, compared to patients who received no counseling from clinical psychologists but who received nutritional and exercise therapy. Above all, this study confirmed that subjects who received counseling lost significantly more weight than those in the control group [4].

Based on our previous findings, the purpose of this study was to identify the psychological factors that promoted behavior modification among the obese patients in this program. The psychological factors were assessed using the Tokyo University Egogram (TEG), which is based on transactional analysis theory.

**Figure 1:**
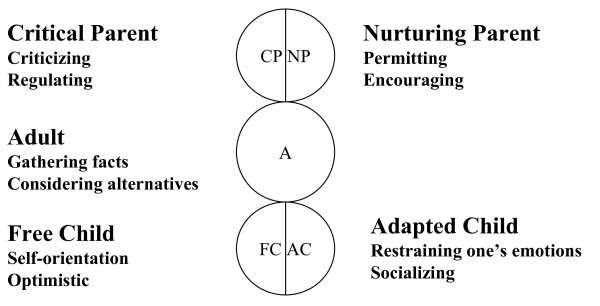
**Ego States**. Berne defined three principal ego states, each with a specific origin and characteristics: Child (archaeopsyche), Parent (exteropsyche), and Adult (neopsyche). Further elaboration of ego state theory led to the recognition of two functional Parent ego states (Critical and Nurturing) and two functional Child ego states (Free and Adapted). CP stands for Critical Parent, with criticizing and regulating characteristics. NP stands for Nurturing Parent, with permitting and encouraging characteristics. A stands for Adult, focusing on gathering facts and considering alternatives and being objective. FC stands for Free Child, with self-orientation and optimistic characteristics. AC stands for Adapted Child, focusing on restraining one's emotions and with social characteristics.

Transactional analysis is a psychological perspective that emphasizes the study of ego states. An ego state is defined as "a coherent system of thoughts and feelings manifested by corresponding patterns of behavior" [5]. Berne defined three principal ego states, each with a specific origin and characteristics: Child (archaeopsyche), Parent (exteropsyche), and Adult (neopsyche) [6]. Further elaboration of ego state theory led to the recognition of two functional Parent ego states (Critical and Nurturing) and two functional Child ego states (Free and Adapted) (Figure 1).

The transactional analysis theory as described above is an effective therapeutic intervention technique that provides an understanding of human behaviors based on observation and measurements.

## Methods and Procedures

### Subjects

A total of 147 (116 female, 31 male) patients participated in a 6-month weight-loss program in our OB clinic between 2002 and 2006. At the start of the weight-loss program, the subjects' mean age was 45.9 ± 15.4 years, their mean weight was 85.1 ± 20.7 kg, and their mean BMI was 33.8 ± 7.0 kg/m^2^.

In accordance with the Declaration of Helsinki and the ethical guidelines established by Kansai Medical University, all study objectives, as well as data protection and analysis methods, were explained to each subject prior to testing, and written informed consent was obtained. All study protocols were approved by the ethics review board of Kansai Medical University.

### Outline of the OB program

**Figure 2:**
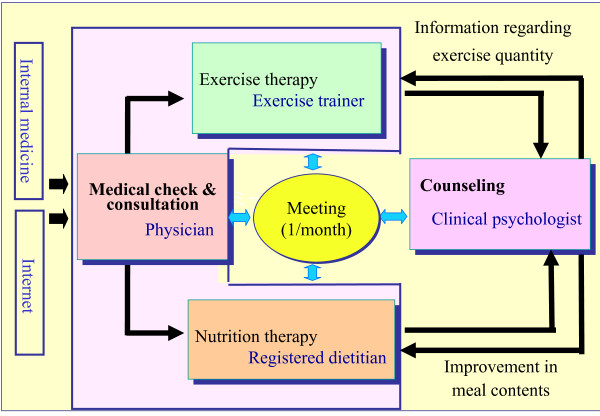
**Obesity program**. The hospital has a 6-month weight-loss program known as the obesity program (OB program). In this program, physicians, clinical psychologists, registered dietitians, and exercise trainers support obese patients. During the 6-month program, obese patients first consulted with a physician for blood and physical examinations. They then participated in intake interviews about their eating habits with a registered dietitian and about their current psychosocial condition with a clinical psychologist. At least once a month, the clinical psychologists met patients for individual face-to-face counseling. A meeting was held every month to discuss methods to support them and to improve their lifestyles.

In the OB program, patients first saw a physician for a medical consultation, and any patients with obesity attributable to endocrine abnormalities or psychiatric disorders were excluded. Subsequently, the patients underwent nutrition and exercise therapy as well as psychological counseling (Figure 2).

As part of the nutrition therapy provided by registered dietitians, the Food Frequency Questionnaire (FFQ) was used to determine the subjects' daily food intake at the start of the program [7]. Subsequently, each subject received individual therapy monthly according to the transtheoretical model [8-10].

As part of the exercise therapy provided by exercise trainers, each subject underwent a cardiopulmonary exercise test as described in our previous report [11]. The anaerobic threshold (AT) was determined, an exercise prescription was made at AT intensity, and then the subjects had exercise therapy in the OB clinic two to three times a month. The exercise therapy lasted for 30 minutes and consisted of both aerobic exercise and stretching. In addition, the subjects were educated about home exercise and received instruction on how to exercise at home: Supervised exercise therapy or non-supervised exercise was performed at least three times a week.

All members of the staff exchanged information on each subject and examined their progress for effective support.

### Intervention by clinical psychologists

First, the clinical psychologists conducted an intake interview for approximately 50 minutes. During the interview, the clinical psychologists listened and determined the patient's psychosocial status in order to better understand their environment and factors associated with obesity. Specifically, psychosocial status refers to the motivation for and expectation of weight loss, social support, and stress. Lifetime changes in weight were also confirmed. During this process, the clinical psychologists identified psychosocial factors that were related to gaining or losing weight.

As part of the subsequent counseling, transactional analysis therapy using the ego-state model and cognitive behavior therapy to improve self-monitoring skills was used. Specifically, the clinical psychologists integrated information about nutrition, exercise, and psychosocial situation, and they supported the subjects so that they could set objectives about diet, exercise, and lifestyle by themselves. The subjects were then instructed to monitor themselves by recording their weight, pedometer counts, and the degree of achievement based on their own objectives. During the psychological counseling, the clinical psychologists tried to focus on relationships among eating habits, physical activity level, psychosocial status, and changes in weight for each of the subjects, as well as focusing on how much success the subject had in achieving their own objectives. It is important to enhance patients' self-effectiveness and self-control in order to reduce psychological stress and to maintain the weight loss. Therefore, the clinical psychologists respected the patients' autonomy and encouraged the patients to set their own objectives on the basis of the dialogic relationship. In addition, stress management techniques and a relaxation method were also adopted when necessary.

### Psychological assessment

During the OB program, psychological characteristics were assessed using several psychological questionnaires. The psychological assessments included the revised NEO personality inventory (NEO-PI-R), which focuses on identifying healthy personality characteristics [12]; the TEG, which measures the ego states [13]; and the Profile of Mood State (POMS), which measures the present mood state [14]. The results from these assessments were then given to the subjects during psychological counseling and used as tools for the subjects to understand how their personalities and stress are related to their eating habits, exercise habits, and weight. Patients answered these questionnaires twice, once when they started the program and once when they completed the program.

In this study, the relationship between the psychological characteristics measured using TEG questionnaires and weight loss was examined. The TEG assessed the energy of each ego state, including Critical Parent (CP), Nurturing Parent (NP), Adult (A), Free Child (FC), and Adapted Child (AC), which are the concepts of the transactional analysis theory. Each ego state has 10 items, so there are 50 items in total.

### Statistics

The data was analyzed using the SPSS12.0J software package. The Wilcoxon signed rank test was used for comparisons of weight and psychological factors before and after intervention. The Mann-Whitney test was used to compare the TEG scores at the start of the program between the subjects who discontinued and those who completed the program. Multiple regression analysis was used to identify factors affecting weight loss. The objective variable was the weight loss during the 6-month program. The explanatory variables were age, the number of sessions of nutrition therapy and counseling, TEG scores at the first session (pre-TEG), and the change in the TEG scores before and after intervention.

## Results

### Change in body weight

Overall, 101 subjects (68.7%) completed the 6-month program. A mean of 6.5 ± 2.3 sessions of nutrition therapy and a mean of 8.0 ± 4.5 counseling sessions were provided to each of the subjects during the program. Exercise therapy was provided at the OB clinic two to three times a month.

The mean weight and BMI at the start of the program of the subjects who completed the program were 83.9 ± 21.0 kg and 33.8 ± 6.9 kg/m^2 ^respectively. The subjects had a statistically significant mean weight loss of 6.2 ± 7.3 kg (Z = 7.72, *p *< 0.01), with weight loss ranging from - 37.6 to 9.2 kg, and a statistically significant mean BMI decrease of 2.4 ± 2.7 kg/m^2 ^(Z = 7.65, *p *< 0.01), with a range from - 12.5 to 3.7 kg/m^2 ^(Table 1).

**Table 1:** Pre and post weight, BMI, and TEG category scores (Wilcoxon signed rank test).

	**Pre**		**Post**			
	***Mean***	***SD***	***Mean***	***SD***	***Z***	***p value***
Weight	83.89	21.00	77.74	18.07	7.72	< 0.01
BMI	33.80	6.85	31.45	6.12	7.65	< 0.01
Critical Parent score	9.78	4.01	10.39	4.08	1.69	0.09
Nurturing Parent score	14.60	4.17	14.46	4.31	0.44	0.66
Adult score	10.09	4.49	10.77	4.78	1.95	0.05
Free Child score	12.00	4.47	12.59	4.64	2.46	0.01
Adapted Child score	9.52	4.62	9.37	4.52	0.25	0.80

The Wilcoxon signed rank test was used for comparisons of weight and psychological factors before and after intervention.

The TEG scores from the first session (pre-TEG) were compared to those taken on completion (post-TEG) of the program in order to identify changes in these patients' psychological characteristics before and after intervention.

### Psychological factors for weight loss

The subjects' scores on psychological testing were: CP, 9.78 ± 4.01; NP, 14.60 ± 4.17; A, 10.09 ± 4.49; FC, 12.00 ± 4.47; and AC, 9.52 ± 4.62. The Mann-Whitney test was used to compare the TEG scores at the start of the program between the subjects who discontinued and those who completed the program; however, no significant differences were observed. We also compared the pre-TEG scores and the change in the TEG scores over six months between men and women by the Mann-Whitney test; however, no significant differences were observed in this comparison either.

Next, the TEG scores from the first session (pre-TEG) were compared to those taken on completion (post-TEG) of the program in order to identify the changes in these patients' psychological characteristics before and after the intervention. Significant differences were observed for the A ego state (0.68 ± 3.56, Z = 1.95, *p *< 0.05) and the FC ego state (0.59 ± 2.74, Z = 2.46, *p *< 0.01) (Table 1). The A ego state is associated with gathering facts and considering alternative characteristics objectively, and the FC ego state is associated with self-orientation and optimistic characteristics. Thus, these characteristics of these subjects appear to have been increased by team medical care intervention.

Furthermore, the multiple regression analysis was performed with weight change as the objective variable and age, the number of sessions of nutrition therapy and counseling, pre-TEG scores, and the change in the TEG scores over six months as explanatory variables. The pre-FC ego state had a positive effect on weight loss (β = 0.33, *p *< 0.01). Thus, the FC ego state, which is related to self-orientation and optimistic characteristics, inhibits weight loss. The A ego state tended to affect weight loss (β = -0.20, *p *= 0.06). Thus, people who improved their A ego state, which is related to gathering facts and considering alternative characteristics, were able to lose more weight (Table 2).

**Table 2:** Factors affecting weight loss (Multiple regression analysis).

**Explanatory variables**	**β coefficient**	***p value***
Pre weight	-0.58	< 0.01
Age	-0.97	0.37
Number of nutrition therapy sessions	0.01	0.93
Number of counseling sessions	0.08	0.37
Pre-Critical Parent score	-0.06	0.67
Pre-Nurturing Parent score	-0.15	0.25
Pre-Adult score	-0.05	0.75
Pre-Free Child score	0.33	0.01
Pre-Adapted Child score	-0.06	0.57
Change in Critical Parent score	0.18	0.09
Change in Nurturing Parent score	-0.15	0.15
Change in Adult score	-0.20	0.06
Change in Free Child score	0.14	0.18
Change in Adapted Child score	0.14	0.17

R(R ^2^)		0.66 (0.43)

The multiple regression analysis was used to identify factors affecting weight loss. The objective variable was the weight loss during the 6-month program. The explanatory variables were age, number of sessions of nutrition therapy and counseling, TEG scores at the first session (pre-TEG), and the changes in TEG score from the first session to the completion of the program.

## Discussion

### Change in body weight

Over the 6-month period, the mean weight loss was 6.2 ± 7.3 kg, and the mean decrease in BMI was 2.4 ± 2.7 kg/m^2^. We previously reported that weight loss can be more easily achieved in a group in which the subjects received counseling in addition to nutrition therapy and exercise therapy [4]. The results of the present study confirmed that this intervention program for obesity resulted in effective weight loss.

The weight loss should be attributed not simply to the intervention of the clinical psychologists but to the total effect of the intervention of a holistic medical care team in which physicians, registered dietitians, exercise trainers, and clinical psychologists were involved as a team. While supporting the results that a comprehensive cognitive behavior modification method using social learning (group approach), self-monitoring, and exercise was most effective [15,16], the present study confirmed the importance of team medical care.

### Psychological factors for weight loss

During the 6-month program, the FC ego state increased significantly; however, the changes in the scores of the FC ego state had no effect on weight loss and the pre-FC ego state was confirmed to prevent weight loss. We examined these results from the perspectives of ego states, each of which have positive and negative aspects. The positive aspects of the FC ego state involve controlling negative emotion and are related to the ability to look on the bright side and do things in one's own style [17], while the negative aspects are not caring about disease and giving in to temptation because of optimism, as well as instinctive and impulsive behaviors. In addition, other studies have reported that some negative emotion has a positive effect on the control of weight and blood sugar levels [4,18]. This study supports these previous findings regarding the relationship between optimism and carelessness in terms of disease prevention behavior modification. Considering the circumstances mentioned above, the negative aspects of the FC ego state prevented subjects from controlling their behavior, while the positive aspects of the FC ego state were increased by team medical care intervention, because the changes in the FC ego state during this program did not prevent weight loss.

The reports by Eysenk, Grossarth-Maticek, and Everitt, and also by Grossarth-Maticek and Eysenk [19,20] also point out that an "object-dependent" personality is a risk factor for disease, and they describe the importance of autonomy training. The enhancement of the patients' FC ego state as related to self-orientation as observed in this study is assumed to indicate that the patients could not only temporarily solve their problems and eliminate certain anxieties, but that they could also change their object-dependent behavior pattern into a more autonomous pattern. Berne [21] said that autonomy resulted from the attainment of three capacities: 1) awareness, 2) spontaneity, and 3) the capacity for intimacy. Joines [22] also stated that the goal of the therapists in transactional analysis is to work themselves out of a job, as the clients are helped to experience their own autonomy and learn to take charge of their own life. That is to say, the result in this study for patients who increased their autonomy shows that the possibility of regaining weight after the program was reduced, whereas the possibility of maintaining the healthy lifestyle obtained through the OB program was increased.

Furthermore, the A ego state increased during the 6-month program and appears to have contributed to weight loss. The A ego state represents self-monitoring skill to get information and determine how well we can do things on the basis of facts. In previous studies, Devin and Reifschneider [15] and Padgett, Mumford, Hynes, and Carter [16] reported that self-monitoring is an important skill for lifestyle modification. In our previous studies, it was also suggested that the A ego state, which involves choosing behaviors suitable for the reality of "here and now," functioning appropriately according to the situation, is associated with improving self-esteem [23,24]. Hence, a person can be more confident in their ability to achieve a healthy lifestyle by taking an approach that allows the A ego state to function appropriately, so that improvement, as well as prevention, of lifestyle-related diseases can be promoted.

### Limitations and future research

In the future, it will be necessary to investigate the factors associated with weight loss in a randomized, controlled trial. In our previous randomized, controlled trial, significant weight loss was observed in a counseling intervention group in which counseling was provided in addition to nutrition and exercise therapy. Therefore, the improvement of autonomy and self-monitoring skills by counseling may have contributed, albeit indirectly, to effectively achieving weight loss.

Moreover, there are other problems, such as the cost and time required to participate in a weight loss program based on a team medical care approach, that need to be resolved in order to reduce the burden on the patient. Given the fact that not all obese patients require counseling, it will also be necessary to establish a system for differentiating between patients who require psychological counseling and those who do not.

Lastly, the present study investigated weight loss and psychological factors for only 6 months; therefore, it will also be useful to examine weight loss and the issue of regaining weight after program completion in a study that includes psychological differences between men and women.

## Conclusion

As a result of the total effect of team medical care involving counseling by clinical psychologists, the A ego state and the FC ego state of obese patients were increased during the 6-month program. Weight loss was observed for patients who had less of an FC ego state at the start of the program and an increased an A ego state during the 6-month program.

## Competing interests

The authors declare that they have no competing interests.

## Authors' contributions

HS conceptualized and designed the study, collected and analyzed the data, performed the statistical analysis, interpreted the results, and drafted the manuscript. YK participated in the design and coordination of the present study and provided clinical data regarding weight. ST contributed to the present study as a nutritionist and NT participated in the present study as an exercise trainer. TB and SS supervised the present study. All authors read and approved the final manuscript.
